# Antiseptic Dressings

**Published:** 1881-03

**Authors:** 


ANTISEPTIC DRESSINGS.—According to *The Pharmaceutical Journal*, the use of carbolic acid for antiseptic dressings is growing more and more in disfavor among the physicians of Germany. In his address before the late Surgeon's Congress in Berlin, on the toxic effect of these carbolic dressings, Dr. Kruester stated that within the last three years he had seen five cases of such poisoning, four of them terminating fatally. He expressed his belief that from the uncertainty of the symptoms shown in such cases, many might have been mistaken as cases of collapse or shock. Professor Koenig, of Gottingen, who at the same Congress advocated permanent irritation of all cases of an already established sepsis of wounds, recommends, exclusively, a solution of salicylic instead of carbolic acid. In the late report of the Berlin University Surgical Clinique, is an account of the death of a child three years old, from an acute carbolism, after the application of carbolic antiseptic dressings, consequent upon an osteotomy of the leg.—*Pittsburg Medical Journal.*

